# The Relation of Intestinal Helminthiasis to Certain Disorders of Vision

**Published:** 1885-09

**Authors:** R. Rampoldi, F. R. Campbell


### The Relation of Intestinal Helminthiasis to Certain Disorders of Vision.

By Dr. R. Rampoldi.

Translated from the Italian in Annali di Ottalmologia, Anno xiv., Fasc. 2 e 3, by F. R. Campbell, M. D.

I have more than once published clinical observations tending to demonstrate the relation existing between the organs of sight and intestinal helminthiasis. In order of time, I first published a clinical history of a case entitled "Retro-retinal cysticercus, anatomically demonstrated by section of the enucleated bulb, tænia solium, present in the same person."

Next, at the meeting of the International Ophthalmological Congress, in Milan, in 1880, I called the attention of the ophthalmologists present to certain accidents to vision, which may be met with in cases of anæmia from anchylostomiasis, namely, accommodative asthenopia, œdema and retinal hemorrhage.

Next, a contribution to the history of "Disorders of vision produced by worms." In this article, I endeavored to give what was then known of the so-called *amblyopia* and *amaurosis verminosa*. I also spoke of reflex *midriasis*, and strabismus convergens, phenomena which, as every one knows, can depend upon irritation produced, primarily, by intestinal worms upon the intestinal mucous membrane, and reflexly upon the nervous centers, as we know that headache, trismus, epilepsy, and even hemiplegia, may be caused in this way.

I have also described some cases of intermittent amblyopia, transitory diplopia and accommodative asthenopia depending upon intestinal worms (ascarides). In one of these cases, photophobia was observed; in another, inequality of the pupils. In all of these cases, the expulsion of the worms was promptly followed by a relief of the symptoms relating to vision. I have recently had a case of complete amaurosis, with extraordinary dilatation of the pupils, and ischæmia of the vasi arteriosi of the retina, cured by the removal of a *tænia solium* from the intestinal tube. I stated, at another time, that these cases had a greater pathological significance when the worms have their seat in the upper part of the alimentary canal.

The facts recently observed by me are, to be sure, not many, but they are not less significant than those formerly given. They are as follows :

1. P. B., a boy, seven years of age, was ill two years ago with diphtheria, at which time he narrowly escaped death. The attack was followed by a secondary paralysis of the pharyngeal muscles, and asthenopia, which soon became a complete paralysis of the muscles of accommodation, so that the nearest point of distinct vision became confused with the remote.

These secondary symptoms were also overcome, and did not re-appear during the following two years. During the first part of January last, the father of the boy observed that the patient opened his eyes in a peculiar manner, closing them spasmodically, and, at the same time, avoiding the light. On being called to see the boy, I found the pupils considerably dilated, and that they did not respond to the light. There was, also, photophobia and blepharospasm. The acuteness of vision was normal, as was also the power of accommodation. The boy was pale and restless. At night, his father said, his sleep was disturbed, and interrupted by sudden spasmodic movements. Suspecting that he was affected with intestinal worms, I gave him a vermifuge and castor oil. After two days, he passed a number of long ascarides, the disorders of vision quickly disappeared, and he went away entirely well.

2. R. E., aged five years, presented at the Ophthalmological Clinic in November, 1883, has suffered with tonic blepharospasm for several days. The energy of the reflex contraction of the lids in this case was quite remarkable. The patient complained of a severe pain over the eyes. As she had some symptoms of intestinal worms, I treated her accordingly, and after she had passed a great number of oxyures and some lumbricoides, she was suddenly found to be well.

During January of this year, the patient was brought back to my clinic with the same symptoms, and, in addition, an inequality of the pupils was observed. I ventured to present the case to my students as an example of the effects of intestinal worms upon vision. The patient was promptly relieved after passing a quantity of oxyures, as before, a:.d five long lumbricoides.

3. B. G., aged 6, brought before the clinic, affected with nystagmus and amblyopia. These symptoms had manifested themselves recently. I at first thought the patient was affected with symptomatic hemeralopia, because the lateral oscillation of the bulb was especially marked, but nothing came to confirm the suspicion. The degree of amblyopia could not well be determined, although it was certain that the acuteness of vision was impaired. There were no visible signs of any alteration at the fundus of the globe. My attention was arrested by the greater dilatation of the left pupil, and I noticed that the eye-ball was more prominent than the right. On learning, from the medical assistant at the clinic, that the child was often restless in his sleep, and at times had convulsions, in which he fell from the bed, I suspected that the disorder might be due to worms. I ordered 8-12 anthelmintic powders alternated with castor oil. The child passed twenty-six lumbricoides, and was immediately cured.

4. G. A., girl, 6 years, attended school for some months, when she was suddenly affected with a *burning* pain in the eyes; there was an uncontrollable desire to shut the eyes, and recurrent amblyopia. Suspecting that the trouble was due to intestinal worms, I ordered vermifuge powders. I learned from the mother that the child, after expelling numerous worms, was cured.

5. While I write these notes (March 7th), I have under observation, at the Ophthalmological Clinic, one Grullo Tomaso, a peasant, 27 years of age, from Ponzone, in whom I have diagnosed cysticercus behind the retina of the left eye, and in whom I have discovered the presence of tænia solium. The demonstration of the cysticercus in the eye will probably be made anatomically in a short time.

I have desired to make a careful record of these cases, because they point out a fact which has been improperly neglected, and the importance of which cannot be overestimated. Without repeated and careful clinical observations, a scientific opinion, such as this, which cannot be demonstrated by experiments and anatomical sections, must be governed entirely by conjecture. It is very evident what an advantage in treatment may be drawn from these observations. I have seen a boy suffering from reflex accommodative asthenopia, due to intestinal worms, treated with iron and quinine for a long time in vain. In another patient, from the same cause, affected with intermittent diplopia, inequality of the pupils, and transitory amblyopia, I have known a diagnosis of progressive degeneration of the cerebral cortex to be made.

To be sure, the number of my observations is not great, but they were all made during the winter months. It is not, however, necessary to assume that functional or organic disorders of vision due to intestinal worms are rare. I remember a boy, who was about to be operated on, cured of a convergent strabismus by the expulsion of numerous ascarides. I recall another case, affected with conjunctival hyperæmia and great photophobia relieved in the same manner. Finally, I observed in April, 1881, at the Dispensary in Pavia, a patient, 19 years of age, who had become blind on account of anæmia from anchylostomiasis. It was learned that this poor man had suffered a long time with "dysentery," and at the time that the repeated and copious hemorrhages occurred, amaurosis was suddenly developed. Sight was irrecoverably lost, and a month after the accident the optic papillæ were of the shining white color so frequently seen in the first stages of cerebral degeneration. Considering the suddenness with which the blindness appeared, that it was associated with atrophy of the optic nerve, and immediately preceded by hemorrhage from the bowels, excited undoubtedly by the presence of intestinal worms (anchylostomata), I maintain that this accident to vision was due to the hemorrhage. Von Graefe was the first to call attention to such a formidable disease of the eye produced in this manner, and, after him, many excellent authorities have reported similar cases.

These cases, we repeat, will remain obscure in their origin, but there is no doubt that there is a real relation between diseased conditions of the primæ viæ and disorders of vision, and that these cases are not merely due to coincidence. In our opinion, there is a difference between cases of amaurosis, which are due to hemorrhage from the alimentary canal, and those which arise from severe anchylostomiasis. We do not deny that there is a condition of general symptomatic anæmia in these latter cases, a condition which may give rise to a so-called lucæmic retinitis first described by Liebreich. It is very easy to find explanations for many other organic and functional diseases of the eye which arise reflexly from the irritation caused by intestinal worms. It is scarcely necessary to mention those cases affected with tænia when an egg from this worm may be transported by the blood to the eye, there to be developed into a cysticercus.

There are plenty of anatomical and physiological explanations for those cases of transitory amblyopia, diplopia, hemiopia, and even of complete amaurosis, due to intestinal helminthiasis.

The pneumo-gastric nerve is the tract through which impulses travel from the organs of digestion to those of sight. The sympathetic, too, is concerned in the production of certain motorial symptoms, such as spasmodic midriasis, projection of the bulb and in one case retraction of the upper lid. Strabismus convergens will have a sufficient explanation, when we recall with Romberg that the external rectus receives a double motor nerve supply (1) from the sixth cranial (patheticus) and (2) from the pneumo-gastric. Asthenopia of accommodation may, according to Remak, be explained in the same manner. Finally, reflex blepharospasm may be excited by the communication existing between the seventh (facial) and tenth (pneumo-gastric) cranial nerves, a communication which the experiments of Bernard have placed beyond doubt. Other experiments made by Inanzi and Lussana have demonstrated that the pneumo-gastric is the sensory and, in a great measure, the excito-secretory nerve of the stomach.

